# Salicylic acid mitigates arsenic-induced toxicity in wheat by enhancing growth and anatomical traits

**DOI:** 10.1080/15592324.2026.2626633

**Published:** 2026-02-10

**Authors:** Usva Ali, Asma Zulfiqar, Ammara Saleem, Muhammad Zafar Saleem, Usman Zulfiqar, Zainul Abideen, Muhammad Awais Arshad, Hossam S. El-Beltagi, Mashael Daghash Alqahtani, Mayank Anand Gururani

**Affiliations:** a Institute of Botany, University of the Punjab, Lahore, Pakistan; b Centre for Applied Molecular Biology, University of the Punjab, Lahore, Pakistan; c Department of Agronomy, Faculty of Agriculture and Environment, The Islamia University of Bahawalpur, Bahawalpur, Pakistan; d Department of Biology, Nakhchivan State University, Nakhchivan, Azerbaijan; e Sharjah, Seed Bank and Herbarium, University of Al Dhaid, Sharjah, United Arab; f Dr. Muhammad Ajmal Khan Institute of Sustainable Halophyte Utilization, University of Karachi, Karachi, Pakistan; g Department of Agronomy, University of Agriculture, Faisalabad, Pakistan; h Agricultural Biotechnology Department, College of Agriculture and Food Sciences, King Faisal University, Al-Ahsa, Saudi Arabia; i Department of Biology, College of Science, Princess Nourah bint Abdulrahman University, Riyadh, Saudi Arabia; j Department of Biology, College of Science, United Arab Emirates University, Al Ain, United Arab Emirates

**Keywords:** Arsenic, salicylic acid, wheat, physiology, anatomy

## Abstract

Salicylic acid (SA) is a key signaling molecule that regulates various physiological and biochemical processes in plants. This study evaluated the role of foliar–applied SA in alleviating arsenic (As) toxicity and improving the growth and anatomical traits of three wheat cultivars (Anaaj-17, Dilkash-20, and Subhani-21) under As stress. Exposure to As (300 and 500 µM) significantly reduced plant height, leaf length, fresh and dry biomass, photosynthetic pigments (chlorophyll *a* and *b*), relative water content, and disrupted leaf and root anatomical structures, including the lower and upper epidermis, cortex, xylem, and phloem thickness. Foliar application of SA (0.5 and 1 mM) mitigated these adverse effects, enhancing growth, chlorophyll content, and vascular and epidermal development in all cultivars. These findings highlight the protective role of SA against As-induced stress, suggesting that its application can improve crop resilience, physiological performance, and anatomical integrity in contaminated soils. The incorporation of SA into crop management practices could contribute to enhanced productivity and sustainable agricultural returns in arsenic-affected areas.

## Introduction

Wheat (*Triticum aestivum* L.), a member of the Poaceae family, is the second most important food crop after rice^1^ and is a staple crop in Pakistan, dominating agricultural production. The country achieved a record production of 27.4 million tons of grain in the 2022–2023 season.^2^ Environmental pollution caused by heavy metals has emerged as a global concern owing to increasing industrialization, urbanization, and anthropogenic activities, which release uncontrolled pollutants into the environment. Wastewater irrigation is a major contributor, leading to the accumulation of heavy metals in soil and plants, ultimately affecting crop productivity and food safety.^3^ Wheat roots readily absorb heavy metals, including arsenic, resulting in accumulation even at low soil concentrations.^4^^,^^5^ This makes wheat particularly vulnerable to arsenic toxicity, which is prevalent in Pakistan, especially in rural Sindh and Punjab.^6^ Arsenic, classified as a Group 1 human carcinogen, exists mainly as arsenate (AsV) and arsenite (AsIII), with arsenate being the most stable form.^7^^-^^9^ WHO guidelines recommend a limit of 0.01 mg L^−^^1^ in drinking water, yet natural water sources often exceed this limit, while permissible soil levels are 24 mg kg^−^^1^.^10^ In affected agricultural regions, soil arsenic may reach 35–46 mg kg^−^^1^.^11^^,^^12^ Arsenic enters plants via phosphate transporters and aquaglyceroporins, disrupting metabolic pathways, photosynthesis, energy production, and redox balance, ultimately impairing growth and nutrient uptake.^13^ Arsenic stress reduces seed germination, root and shoot elongation, biomass, and essential nutrients such as Cu, Fe, N, K, and P.^13^ It also affects plant anatomy by altering root and leaf structures, membrane permeability, and vascular development, contributing to impaired water and ion transport and stunted growth.

Plant growth regulators, particularly salicylic acid (SA), play a pivotal role in alleviating heavy metal toxicity and enhancing abiotic stress tolerance. SA, a phenolic compound derived from Salix (Latin for willow tree), regulates multiple physiological and biochemical processes, including thermogenesis, signaling, defense mechanisms, and responses to both biotic and abiotic stressors.^14^^,^^15^ SA influences plant morphology, development, flowering, stomatal behavior, and photosynthetic activity.^16^ Foliar application of SA has been shown to improve growth, leaf number, biomass, and stem diameter in wheat,^17^ while low concentrations enhance pigment content and antioxidant capacity, mitigating heavy metal-induced oxidative damage.^18^ Anatomical studies indicate that abiotic stress reduces epidermal and cortical thickness, vascular bundle size, and mesophyll layers, limiting water and nutrient transport and reducing photosynthetic efficiency.^19^^,^^20^ Conversely, exogenous SA application positively affects anatomical traits, including upper and lower epidermis, cortex, xylem, and phloem development, improving hydraulic conductivity, nutrient translocation, and stress resilience.^21^ SA-mediated enhancement of root and leaf anatomy helps plants maintain structural integrity under stress, which is essential for sustainable growth and yield. This aspect is particularly important in wheat, where leaf and root anatomical modifications directly influence the photosynthetic capacity, nutrient uptake, and overall productivity under heavy metal stress.

The objective of this study was to investigate the detrimental effects of arsenic on wheat anatomical features (upper and lower epidermis, vascular bundles, and cortex) and physiological traits (chlorophyll *a*, *b,* and relative water content) and to evaluate the mitigating role of foliar–applied SA. We hypothesized that SA application enhances the anatomical and physiological features of wheat under arsenic stress, thereby improving stress tolerance and supporting sustainable crop production.

## Materials and methods

### Experimental material and plan

**Figure 1.**
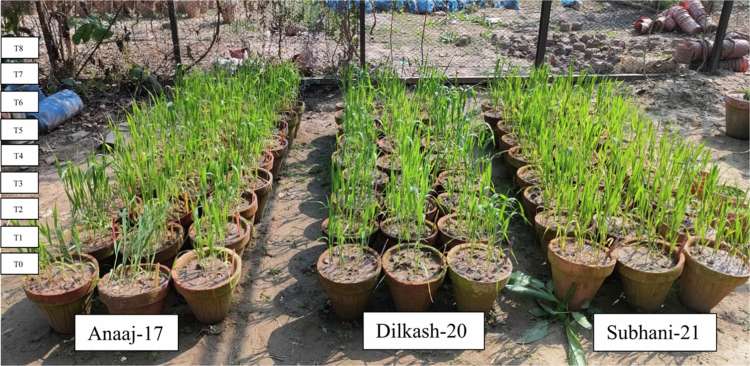
Experimental setup showing labeled pots of wheat cultivars under different treatments. Groups one, two, and three represent the cultivars Anaaj-17, Dilkash-20, and Subhani-21, respectively. Each treatment was labeled as T0–T8, and each treatment included three biological replicates. Experimental setup in wirehouse no. 06 at Botanical Garden University of Punjab, Lahore. (T0) control; (T1) 0.5 mM SA + 0 μM As; (T2) 1 mM SA + 0 μM As; (T3) 0 mM SA + 300 μM As; (T4) 0 mM SA + 500 μM As; (T5) 0.5 mM SA + 300 μM As; (T6) 0.5 mM SA + 500 μM As; (T7) 1 mM SA + 300 μM As; (T8) 1 mM SA + 500 μM As.

Three wheat cultivar seeds, Anaaj-17, Dilkash-20, and Subhani-21, were collected from the Federal Seed Certification and Registration Department. The sterilization process involved treating the seeds with sodium hypochlorite (NaClO) for 15–20 min to eliminate any potential pathogens on their surfaces. Following sterilization, the seeds were soaked overnight in water to initiate the germination process. When 2–3 leaves appeared, treatments were applied to the plants. Two salicylic acid (SA) concentrations (0.5 and 1 mM) and two arsenic (As) concentrations (300 and 500 µM) were selected based on prior work^22^^,^^23^ and combined to generate the following nine treatments (T0–T8). The treatments used were as follows: (T0) 0 mM SA + 0 μM As (control), (T1) 0.5 mM SA + 0 μM As, (T2) 1 mM SA + 0 μM As, (T3) 0 mM SA + 300 μM As, (T4) 0 mM SA + 500 μM As, (T5) 0.5 mM SA + 300 μM As, (T6) 0.5 mM SA + 500 μM As, (T7) 1 mM SA + 300 μM As, and (T8) 1 mM SA + 500 μM As (Figure 1). The source of arsenic was hydrated sodium arsenate (Na_2_HAsO_4_·7H_2_O). Fifteen weeks after sowing, plant specimens were collected to measure plant growth attributes (plant height/plant, fresh weight/plant, and dry weight/plant) and physiological characteristics (chlorophyll *a*, chlorophyll *b*, chlorophyll *a/b*, and relative water content) and anatomical parameters (upper epidermis thickness, lower epidermis thickness, cortex thickness, xylem thickness, phloem thickness, mesophyll thickness, and vascular bundle thickness).

### Measurement of growth characteristics

The height of the plant was recorded directly using a meter scale. The fresh weight (FW) of each sample was determined by placing them in an oven set to 60 °C for drying. After 48 h, the dry weights of the samples were measured.

### Determination of physiological parameters

We utilized fresh leaves from each plant for every replicate and treatment for total chlorophyll estimation. Each 0.2 g leaf sample was immersed in 10 ml of DMSO in Falcon tubes. The leaves were incubated in the dark for 48 h at 25 °C ± 2 °C. The absorbance of the extracts was measured using a spectrophotometer at 645 and 663 nm.

The total chlorophyll content (µg/g) was determined using the formula of Arnon et al.^24^:

The total chlorophyll content = [(0.0082) × (D-663) + (0.0202) × (D-645)] × (mL of solvent)/fresh weight of leaves (grams).

The relative water content (RWC) of each leaf was measured at each harvest using three fully expanded leaves per plant per pot, following Barrs and Weatherley.^25^ Detached leaf samples were immediately weighed to determine the fresh weight (FW) and then placed in covered Petri plates with water for full hydration. After 18 h at 4 °C, the leaves were blotted dry and weighed to determine the turgid weight (TW). The leaf tissue was then dried in an oven at 75 °C for 72 h to determine dry weight (DW).

- The relative water content was found using the formulation:

RWC(%)=[(Wf−Wd)/(Wt−Wd)]×100.

### Anatomical studies

For anatomical analysis, the plants were preserved in a formalin acetic acid solution consisting of 80% alcohol, 10% formalin, and 10% acetic acid. Following a 24-h preservation period, the roots and leaves of the uprooted and washed wheat cultivars were cut into pieces and placed in labeled bottles. Using the freehand technique, a transverse section was cut from the stored specimens and transferred to a clean slide containing a drop of water. A solution series of 10%, 30%, 50%, 70%, 90%, and 100% alcohol, along with safranin in 70% alcohol, was prepared for serial dehydration. A drop of 50% safranin was added to the slide to stain the section, and the specimen was covered with a coverslip. The internal structures were observed and measured under a camera-fitted compound light microscope NIOF X8Z-107BN.^26^ The ImageJ program was used for measuring the sections.

### Statistical analysis

A two-way analysis of variances (ANOVA) was used to compare the means, and the least significant difference (LSD) test was used to determine significant differences among the individual means of the treatments and cultivars.

## Results

### Changes in growth parameters

#### Effect of SA on the morphology of wheat cultivars under As stress

**Figure 2.**
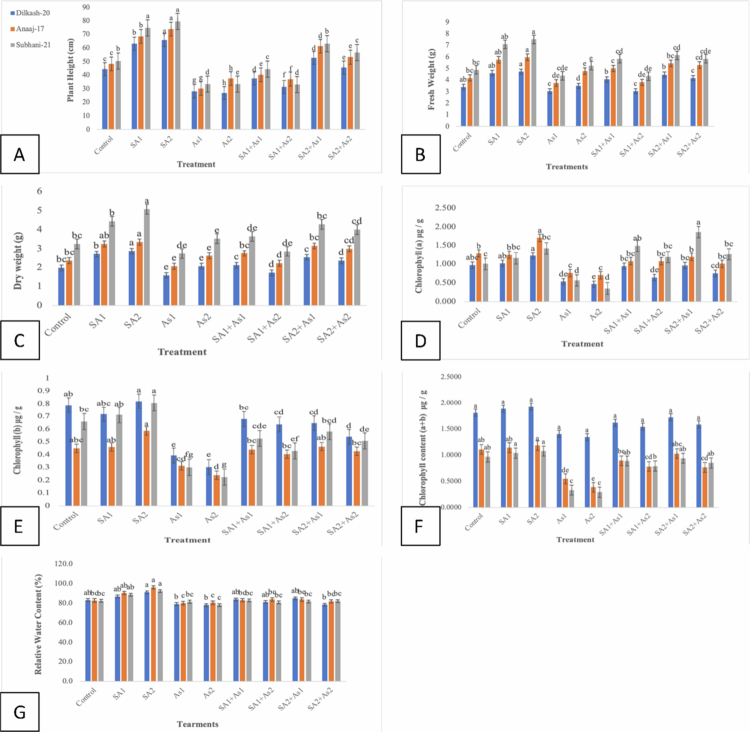
Effect of SA (0.5 mM, 1 mM) on selected *T. aestivum* cultivars under arsenic stress (300 μM and 500 μM). Plant height (A), fresh weight (B), dry weight (C), chlorophyll *a* (D), *b* (E), total chlorophyll (F), and relative water content (G). According to LSD, bars with distinct alphabet letters are substantially (*p* < 0.05) different from one another. All the values in the graph are the mean of triplicates.

Plants of all *T. aestivum* cultivars, after treatment with different levels of SA via foliar application, showed better plant growth with higher plant height and weight. Figure 2(A–C) displays various morphological characteristics of *T. aestivum* cultivars. The study's findings showed that plant height, fresh weight, and dry weight per plant increased by increasing SA concentrations (0.5 mM (SA1) and 1 mM (SA2)) than those of the control plants. On the other hand, growing concentration of arsenic stress (300 μM (As1) and 500 μM (As2)) led to a reduction. Combination treatments revealed complex interactions, with 1 mM salicylic acid and 300 μM arsenic (SA2 + As1), showing a notable synergistic effect in Figure 2(A–C), resulting in a substantial recovery of fresh weight and dry weight compared to individual arsenic treatments.

#### Effect of SA on photosynthetic pigments and relative water content of wheat cultivars under As stress

Salicylic acid (SA) treatments generally increase chlorophyll content across all the wheat cultivars, with the 1 mM (SA2) concentration being more effective. Arsenic (As) treatments reduce chlorophyll content, with a higher concentration of arsenic having a more pronounced negative effect. The combined salicylic acid and arsenic treatments showed a mitigating effect, particularly with SA2 combined with arsenic. The result regarding the photosynthetic pigment is presented in Figure 2(D–F). The combination of 1 mM salicylic acid with 500 μM arsenic (T8: SA2+As2) showed a mixed recovery, with chl-*a* content of 0.757 for Dilkash-20, 1.007 for Anaaj-17, and 1.267 for Subhani-21. Chl-*b* content was significantly increased with the same pattern as in chl-*a*, reaching the highest value with SA2. Still, the chl-*b* content was higher at each concentration in all the cultivars (Figure 2E). The treatment with SA2 (1 mM salicylic acid) alone provided the highest total chlorophyll content across all three wheat cultivars (Figure 2F). Arsenic treatments significantly reduced the total chlorophyll content, and the combined treatments of salicylic acid and arsenic showed a mitigation effect, particularly with SA2 combined with arsenic.

The relative water content (RWC) of three wheat cultivars, Anaaj-17, Dilkash-20, and Subhani-21, varied under the different treatments. The control group (T0) showed relatively high RWC values, with 83.1% for Dilkash-20, 82.7% for Anaaj-17, and 82.3% for Subhani-21. Treatments with salicylic acid (SA) alone increased the RWC across all the wheat cultivars, as shown in Figure 2G. On the other hand, decreases in RWC occurred when the plants received the arsenic treatments, As1 at 300 μM and As2 at 500 μM. In the case of the As1 treatment, the obtained RWC values were 79.0% for Dilkash-20, 80.4% for Anaaj-17, and 81.3% for Subhani-21. The As2 treatment also reduced the RWC to 78.0% for both the Dilkash-20 and Subhani-21, while Anaaj-17 reached 80.3%.

#### Changes in the anatomical characteristics of leaves

**Figure 3.**
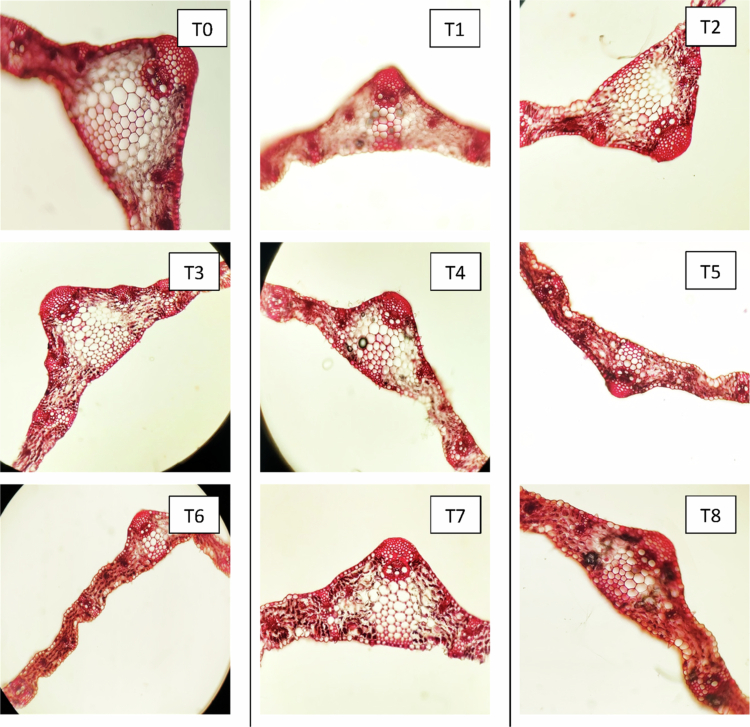
Cross section of a wheat leaf (Subhani-21) grown under arsenic stress. (T0) control; (T1) 0.5 mM SA  + 0 μM As; (T2) 1 mM SA + 0 μM As; (T3) 0 mM SA + 300 μM As; (T4) 0 mM SA + 500 μM As; (T5) 0.5 mM SA + 300 μM As; (T6) 0.5 mM SA + 500 μM As; (T7) 1 mM SA + 300 μM As; and (T8) 1 mM SA + 500 μM As.

**Figure 4.**
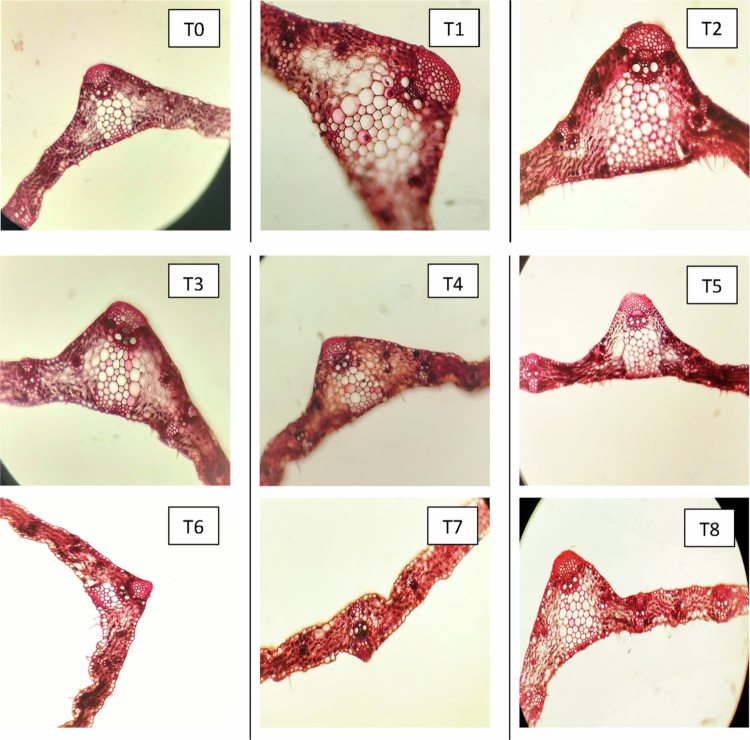
Cross section of a wheat leaf (Annaj-17) grown under arsenic stress. (T0) control; (T1) 0.5 mM SA + 0 μM As; (T2) 1 mM SA + 0 μM As; (T3) 0 mM SA + 300 μM As; (T4) 0 mM SA + 500 μM As; (T5) 0.5 mM SA + 300 μM As; (T6) 0.5 mM SA + 500 μM As; (T7) 1 mM SA + 300 μM As; and (T8) 1 mM SA + 500 μM As.

**Figure 5.**
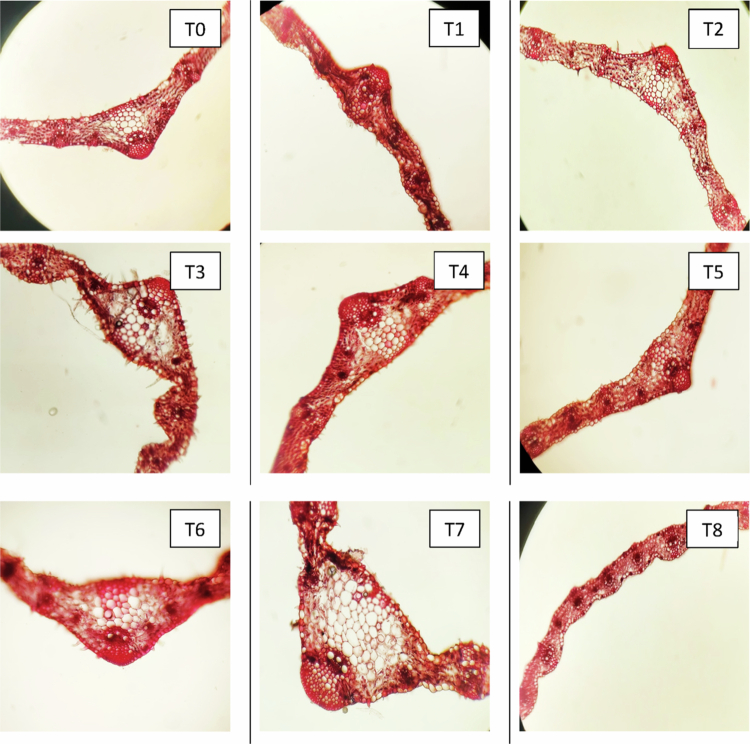
Cross section of a wheat leaf (Dilkash-20) grown under arsenic stress. (T0) control; (T1) 0.5 mM SA + 0 μM As; (T2) 1 mM SA + 0 μM As; (T3) 0 mM SA + 300 μM As; (T4) 0 mM SA + 500 μM As; (T5) 0.5 mM SA + 300 μM As; (T6) 0.5 mM SA + 500 μM As; (T7) 1 mM SA + 300 μM As; and (T8) 1 mM SA + 500 μM As.

Anatomical sections of the leaves from three distinct wheat cultivars—Subhani-21, Anaaj-17, and Dilkash-20—are depicted in Figure 3, Figure 4, and Figure 5, respectively. The analysis revealed that different treatments have varying and significant impacts on leaf anatomy. Remarkably, alterations in the thickness of the epidermis, mesophyll, and vascular bundles were observed across the cultivars under the influence of salicylic acid (SA) and arsenic (As) treatments. These anatomical adjustments highlight the adaptive strategies of plants to cope with arsenic stress and underscore the beneficial effects of salicylic acid. The plants treated with SA exhibited enhanced anatomical characteristics, indicating a substantial improvement in their resilience to arsenic toxicity. The fully labeled microscopic images of the leaf anatomical structures are provided in the Supplementary File (Supplementary Figure S1).

#### Effect of SA on the lower epidermis of leaves of wheat cultivars under As stress

**Figure 6.**
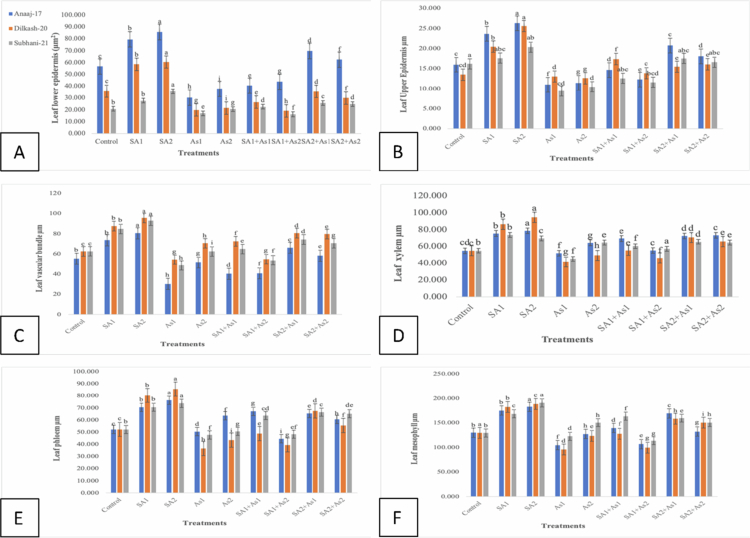
Effect of SA (0.5 mM and 1 mM) on selected *T. aestivum* cultivars under arsenic stress (300 μM and 500 μM). Leaf lower epidermis (A), leaf upper epidermis (B), leaf vascular bundle (C), leaf xylem (D), leaf phloem (E), and leaf mesophyll (F). According to LSD, bars with distinct alphabet letters are substantially (*p* < 0.05) different from one another. All the values in the graph are the mean of triplicates.

The lower epidermis (LE) thickness of three wheat cultivars, Anaaj-17, Dilkash-20, and Subhani-21, showed significant variations under the different treatments. The control conditions (T0) revealed the highest LE thickness in Anaaj-17 (56.693 µm) and the lowest in Subhani-21 (20.748 µm). Treatments with salicylic acid alone, particularly SA2 (1 mM), markedly increased LE thickness across all cultivars, with Anaaj-17 reaching 85.683 µm, Dilkash-20 at 60.393 µm, and Subhani-21 at 35.637 µm. In contrast, the arsenic treatments (As1 at 300 μM and As2 at 500 μM) significantly reduced the LE thickness, with the lowest values observed in Dilkash-20 (19.818 µm under As1) and Subhani-21 (16.895 µm under As1). Combined treatments of salicylic acid and arsenic generally mitigated the adverse effects of arsenic alone, with SA2 + As1 showing a substantial recovery in LE thickness for Anaaj-17 (69.737 µm) and Subhani-21 (25.633 µm), as shown in Figure 6A.

#### Effect of SA on the upper epidermis of leaves of wheat cultivars under As stress

Wheat under arsenic stress showed a decrease in diameter, as compared to the control. The effects of various treatments on the upper epidermis (UE) thickness of three wheat cultivars, Anaaj-17, Dilkash-20, and Subhani-21, revealed significant differences. Under control conditions (T0), the UE thickness was highest for Subhani-21 (16.198 µm) and lowest for Dilkash-20 (13.490 µm). Treatment with salicylic acid alone, particularly SA2 (1 mM), significantly increased UE thickness across all cultivars, reaching 26.287 µm for Anaaj-17, 25.600 µm for Dilkash-20, and 20.347 µm for Subhani-21. In contrast, arsenic treatments (As1 at 300 μM and As2 at 500 μM) decreased the UE thickness, with the lowest values observed in Anaaj-17 (10.988 µm under As1) and Subhani-21 (9.435 µm under As1). The combined salicylic acid and arsenic treatments had varying effects, generally mitigating the reduction caused by arsenic alone. For instance, the SA2+As1 combination resulted in 20.777 µm for Anaaj-17 and 17.533 µm for Subhani-21, indicating a partial recovery. Overall, salicylic acid treatments enhanced UE thickness, while arsenic treatments reduced it, with the combined treatments showing an intermediate effect, as shown in Figure 6B.

#### Effect of SA on the vascular bundle thickness of leaves of wheat cultivars under As stress

The vascular bundle (VB) thickness in three wheat cultivars, Anaaj-17, Dilkash-20, and Subhani-21, showed significant variations under the different treatments. Under control conditions (T0), the VB thickness was similar across cultivars, with values around 55.31 µm for Anaaj-17, 62.64 µm for Dilkash-20, and 62.68 µm for Subhani-21. Treatments with salicylic acid alone, particularly SA2 (1 mM), significantly increased the VB thickness, reaching up to 80.54 µm in Anaaj-17, 95.61 µm in Dilkash-20, and 92.9 µm in Subhani-21. Arsenic treatments (As1 at 300 μM and As2 at 500 μM) reduced the VB thickness, with the most pronounced decrease in Anaaj-17 (30.22 µm under As1), as shown in Figure 6C. Combined treatments of salicylic acid and arsenic mitigated the negative impact of arsenic, with the SA2 + As1 combination resulting in substantial recovery of the VB thickness, particularly in Anaaj-17 (66.2 µm) and Dilkash-20 (80.70 µm).

#### Effect of SA on the xylem thickness of leaves of wheat cultivars under As stress

The xylem thickness (*X*) in three wheat cultivars, Anaaj-17, Dilkash-20, and Subhani-21, displayed significant changes under various treatments. Under control conditions (T0), the xylem thickness was consistent across all cultivars, around 55 µm. Treatment with salicylic acid, particularly SA2 (1 mM), resulted in a notable increase in xylem thickness, with values reaching 78.667 µm for Anaaj-17, 94.550 µm for Dilkash-20, and 69.577 µm for Subhani-21. Arsenic treatments (As1 at 300 μM and As2 at 500 μM) generally led to a reduction in xylem thickness, especially for Dilkash-20 (41.608 µm under As1) and Anaaj-17 (52.040 µm under As1). However, combined treatments of salicylic acid and arsenic helped mitigate the adverse effects of arsenic alone. For instance, the SA2 + As1 treatment resulted in substantial recovery of xylem thickness, particularly for Anaaj-17 (72.330 µm) and Dilkash-20 (70.660 µm). Overall, the salicylic acid treatments enhanced xylem thickness, whereas the arsenic treatments reduced it, and the combined treatments demonstrated an intermediate effect, partially alleviating the negative impact of arsenic, as shown in Figure 6D.

#### Effect of SA on the phloem thickness of leaves of wheat cultivars under As stress

The phloem thickness (Ph) of three wheat cultivars, Anaaj-17, Dilkash-20, and Subhani-21, exhibited noticeable differences under various treatments. Under control conditions (T0), the phloem thickness was approximately 52 µm across all the cultivars. Salicylic acid treatments, particularly SA2 (1 mM), significantly increased phloem thickness, reaching up to 76.397 µm in Anaaj-17, 85.567 µm in Dilkash-20, and 73.760 µm in Subhani-21. Arsenic treatments (As1 at 300 μM and As2 at 500 μM) generally reduced phloem thickness, with the most notable reduction observed in Dilkash-20 (36.590 µm under As1). The combined salicylic acid and arsenic treatment generally mitigated the negative impact of arsenic alone. The SA2 + As1 combination resulted in relatively high phloem thickness, particularly in Dilkash-20 (67.460 µm) and Anaaj-17 (65.363 µm), as shown in Figure 6E.

#### Effect of SA on the mesophyll thickness of leaves of wheat cultivars under As stress

The mesophyll thickness (*M*) in three wheat cultivars, Anaaj-17, Dilkash-20, and Subhani-21, varied significantly under different treatments. Under control conditions (T0), mesophyll thickness was similar across cultivars, around 130 µm. Treatments with salicylic acid alone, particularly SA2 (1 mM), greatly increased mesophyll thickness, with Anaaj-17 reaching 182.740 µm, Dilkash-20 at 188.673 µm, and Subhani-21 at 190.753 µm. The arsenic treatments (As1 at 300 μM and As2 at 500 μM) generally reduced the mesophyll thickness, with the lowest values in Dilkash-20 (95.683 µm under As1) and Anaaj-17 (104.633 µm under As1). The combined salicylic acid and arsenic treatments showed mixed effects, often mitigating the reduction caused by arsenic. Notably, SA2 + As1 treatment significantly recovered mesophyll thickness, particularly for Anaaj-17 (169.350 µm) and Dilkash-20 (158.513 µm), as summarized in Figure 6F.

#### Changes in the anatomical characteristics of roots

**Figure 7.**
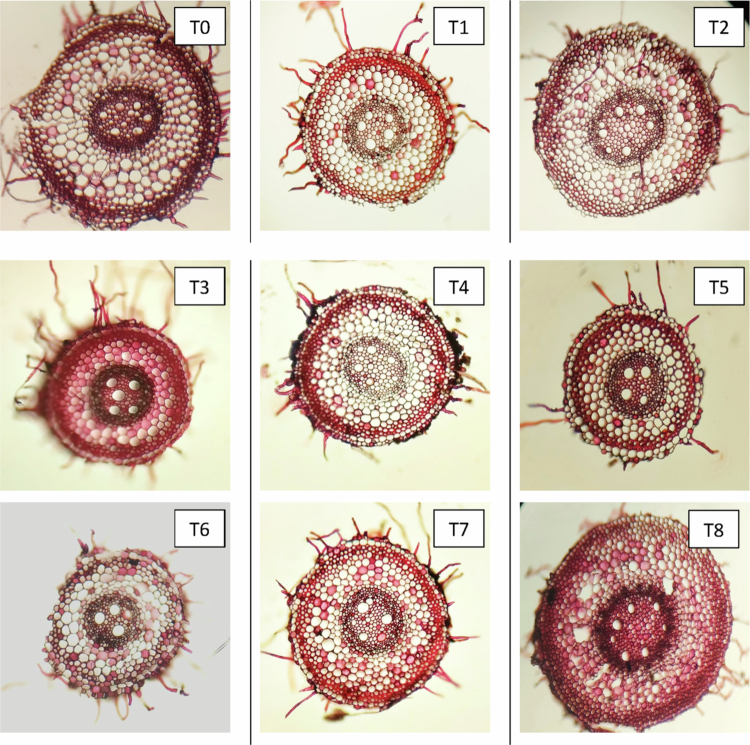
Cross section of wheat root (Subhani-21) grown under arsenic stress. (T0) control; (T1) 0.5 mM SA + 0 μM As; (T2) 1 mM SA + 0 μM As; (T3) 0 mM SA + 300 μM As; (T4) 0 mM SA + 500 μM As; (T5) 0.5 mM SA + 300 μM As; (T6) 0.5 mM SA + 500 μM As; (T7) 1 mM SA + 300 μM As; and (T8) 1 mM SA + 500 μM As.

**Figure 8.**
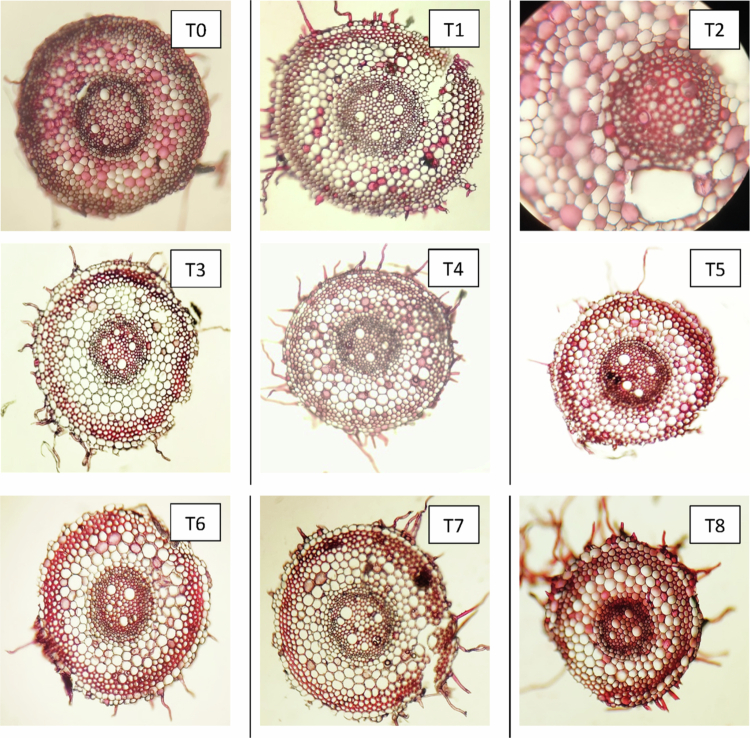
Cross section of wheat root (Anaaj-17) grown under arsenic stress. (T0) control; (T1) 0.5 mM SA + 0 μM As; (T2) 1 mM SA + 0 μM As; (T3) 0 mM SA + 300 μM As; (T4) 0 mM SA + 500 μM As; (T5) 0.5 mM SA + 300 μM As; (T6) 0.5 mM SA + 500 μM As; (T7) 1 mM SA + 300 μM As; and (T8) 1 mM SA + 500 μM As.

**Figure 9.**
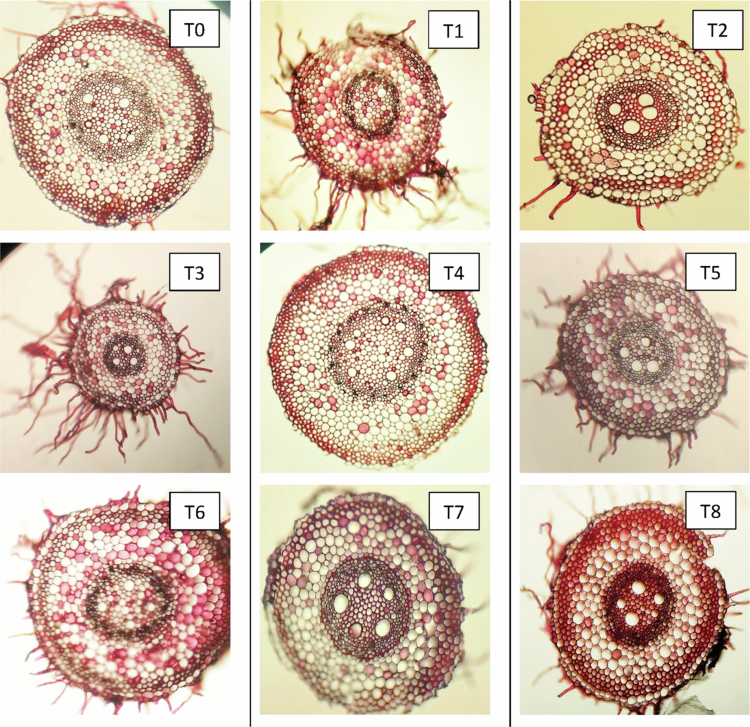
Cross section of wheat root (Dilkash-20) grown under arsenic stress. (T0) control; (T1) 0.5 mM SA + 0 μM As; (T2) 1 mM SA + 0 μM As; (T3) 0 mM SA + 300 μM As; (T4) 0 mM SA + 500 μM As; (T5) 0.5 mM SA + 300 μM As; (T6) 0.5 mM SA + 500 μM As; (T7) 1 mM SA + 300 μM As; and (T8) 1 mM SA + 500 μM As.

Anatomical sections of the roots from three distinct wheat cultivars—Subhani-21, Anaaj-17, and Dilkash-20—are illustrated in Figure 7, Figure 8, and Figure 9, respectively. The analysis indicates significant differences in root anatomy among the cultivars under various treatments. Notably, factors such as root thickness, the arrangement of vascular tissues, and overall root structure were influenced by treatments involving salicylic acid and arsenic. The fully labeled microscopic images of the root anatomical structures are provided in the Supplementary File (Supplementary Figure S1).

### Effect of SA on the exodermis thickness of roots, cortex of roots, xylem, and phloem thickness of wheat cultivars under As stress

**Figure 10.**
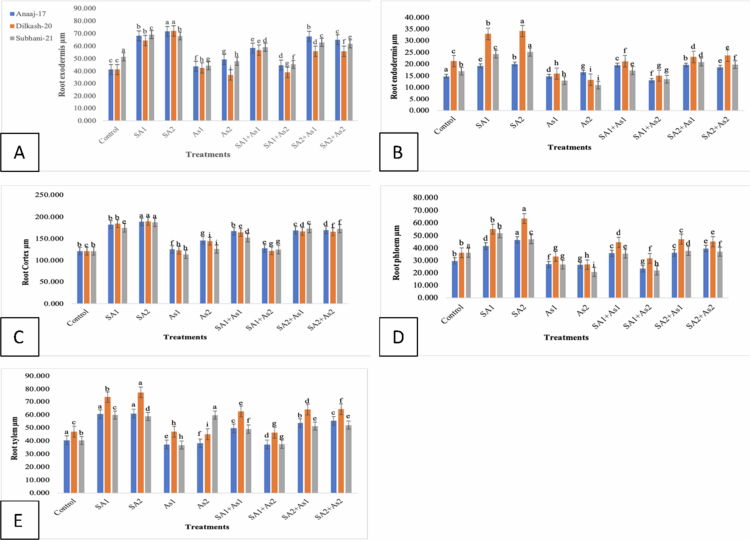
Effect of SA (0.5 mM and 1 mM) on selected *T. aestivum* cultivars under arsenic stress (300 μM and 500 μM). Root exodermis (A), root endodermis (B), root cortex (C), root phloem (D), and and root xylem (E). According to LSD, bars with distinct alphabet letters are substantially (*p *< 0.05) different from one another. All the values in the graph are the mean of triplicates.

The exodermis thickness (Ex) of the roots in three wheat cultivars, Subhani-21, Anaaj-17, and Dilkash-20, varied significantly under the different treatments. Under control conditions (T0), the exodermis thickness was highest in Subhani-21 (51.517 µm) and similar for Anaaj-17 (41.483 µm) and Dilkash-20 (41.360 µm). Salicylic acid treatments, particularly SA2 (1 mM), significantly increased the thickness of the exodermis across all cultivars, reaching up to 71.863 µm in Anaaj-17, 72.177 µm in Dilkash-20, and 69.333 µm in Subhani-21. The arsenic treatments (As1 at 300 μM and As2 at 500 μM) generally resulted in a lower exodermis thickness than salicylic acid treatments, with the lowest values observed in Dilkash-20 (37.003 µm under As2). The combined salicylic acid and arsenic treatments showed intermediate effects, mitigating the reduction caused by arsenic alone. For instance, the SA2 + As1 treatment resulted in substantial recovery, especially for Anaaj-17 (67.900 µm) and Subhani-21 (63.123 µm), as shown in Figure 10A. The endodermis characteristics (En) of three wheat cultivars, Anaaj-17, Dilkash-20, and Subhani-21, were notably influenced by different treatments. The control conditions (T0) exhibited varying En values among the cultivars, with Subhani-21 displaying the highest value (17.000), followed by Dilkash-20 (21.337) and Anaaj-17 (14.817). Salicylic acid treatments, especially SA2 (1 mM), significantly increased the En values across all the cultivars, peaking at 25.173 for Subhani-21, 34.217 for Dilkash-20, and 20.050 for Anaaj-17, as shown in Figure 10B. Conversely, arsenic treatments (As1 at 300 μM and As2 at 500 μM) generally led to lower En values compared to salicylic acid treatments, with the lowest values observed under As2 treatment. The combined salicylic acid and arsenic treatments displayed intermediary effects, partially alleviating the reduction caused by arsenic alone. For instance, the SA2 + As1 treatment resulted in notable recovery, particularly for Anaaj-17 (17.237) and Subhani-21 (19.627). The cortex (C) characteristics of three wheat cultivars, Anaaj-17, Dilkash-20, and Subhani-21, were significantly influenced by different treatments. Under control conditions (T0), the cortex values were relatively similar across all the cultivars. Salicylic acid treatments, particularly SA2 (1 mM), notably increased the cortex values across all cultivars, reaching peaks of 188.790 for Anaaj-17, 189.630 for Dilkash-20, and 187.347 for Subhani-21. The arsenic treatments generally resulted in lower cortex values compared to salicylic acid treatments, with the lowest values observed under the As2 treatment, particularly in Subhani-21 (126.403). The combined salicylic acid and arsenic treatments exhibited intermediary effects, partially alleviating the reduction caused by arsenic alone. For instance, the SA1+As1 treatment showed moderate recovery, especially for Anaaj-17 (167.400) and Subhani-21 (152.253), as summarized in Figure 10C. The phloem (Ph) characteristics of three wheat cultivars, Anaaj-17, Dilkash-20, and Subhani-21, demonstrated notable variations under different treatments. Salicylic acid treatments, particularly SA2 (1 mM), notably increased phloem values across all cultivars, with peaks observed for Dilkash-20 (63.610), Anaaj-17 (46.583), and Subhani-21 (47.090). The arsenic treatments generally resulted in lower phloem values compared to salicylic acid treatments, with the lowest values recorded under the As2 treatment, especially for Subhani-21 (20.663). Combined salicylic acid and arsenic treatments exhibited intermediary effects, partially alleviating the reduction caused by arsenic alone. For instance, the SA1+As1 treatment displayed moderate recovery, particularly for Dilkash-20 (44.560) and Subhani-21 (35.597), as summarized in Figure 10D. The xylem (X) characteristics of three wheat cultivars, Anaaj-17, Dilkash-20, and Subhani-21, demonstrated notable variations under different treatments. The salicylic acid treatments, particularly SA2 (1 mM), notably increased the xylem values across all the cultivars, with peaks observed for Dilkash-20 (77.417), Anaaj-17 (60.977), and Subhani-21 (58.790). The arsenic treatments generally resulted in varied xylem values compared to salicylic acid treatments, with differing impacts observed across cultivars. Combined treatments of salicylic acid and arsenic showed intermediary effects, with varying degrees of recovery compared to arsenic treatments alone. For instance, the SA2+As1 treatment displayed moderate recovery, particularly for Anaaj-17 (53.620) and Dilkash-20 (64.087), as shown in Figure 10E. Overall, the salicylic acid treatments tended to enhance the xylem characteristics, while arsenic treatments had varying effects, and the combined treatments exhibited a mitigating impact, albeit with differences across cultivars.

#### Effect of salicylic acid on antioxidant enzyme activities under arsenic stress

The activities of the antioxidant enzymes superoxide dismutase (SOD) and peroxidase (POD) were significantly influenced by salicylic acid (SA) and arsenic (As) treatments in all three wheat varieties (Anaaj-17, Dilkash-20, and Subhani-21) (Table 1). Exposure to arsenic alone (T3 and T4) caused a marked increase in both SOD and POD activities compared to the control, indicating activation of the plant's defense system in response to oxidative stress. Notably, the application of SA, either alone (T1 and T2) or in combination with arsenic (T5–T8), further enhanced the activities of these antioxidant enzymes. The highest SOD and POD levels were observed under the combined SA + As treatments, suggesting that SA alleviates arsenic-induced phytotoxicity by stimulating the antioxidative defense mechanism of plants.

**Table 1.** Effects of exogenous salicylic acid (SA) on superoxide dismutase (SOD) and peroxidase (POD) activities in three wheat cultivars under varying arsenic (As) stress levels.

SOD				POD		
Treatments	Anaaj-17 (units/mg protein)	Dilkash-20 (units/mg protein)	Subhani-21 (units/mg protein)	Anaaj-17 (units/mg protein)	Dilkash-20 (units/mg protein)	Subhani-21 (units/mg protein)
T0 (control)	10.50d	8.20d	9.15d	4.20d	3.50d	3.80d
T1 (SA1)	13.10c	11.55c	12.40c	5.50c	4.85c	5.10c
T2 (SA2)	14.20c	12.65c	13.50c	6.15c	5.40c	5.75c
T3 (As1)	22.75b	20.10b	21.50b	9.80b	8.60b	9.15b
T4 (As2)	25.90b	23.45b	24.80b	11.50b	10.25b	10.80b
T5 (SA1+As1)	28.15a	25.90a	27.35a	13.20a	11.90a	12.55a
T6 (SA1+As2)	30.50a	28.33a	29.70a	15.00a	13.50a	14.25a
T7 (SA2+As1)	29.30a	27.10a	28.55a	14.15a	12.80a	13.40a
T8 (SA2+As2)	31.85a	29.50a	30.90a	16.50a	15.00a	15.80a

Note: Means followed by different lowercase letters within a column are significantly different at *p* ≤ 0.05 according to LSD test.

#### Effect of salicylic acid on oxidative stress markers under arsenic stress

Table 2 shows the effect of salicylic acid (SA) on the oxidative stress markers, malondialdehyde (MDA) and hydrogen peroxide (H₂O₂), in three wheat varieties (Anaaj-17, Dilkash-20, and Subhani-21). The arsenic treatments (T3 and T4) markedly increased the MDA level by 746%–900% and the H₂O₂ level by 1150%–1220% compared to the control, indicating severe oxidative stress. Application of SA, either alone (T1 and T2) or combined with arsenic (T5–T8), significantly reduced these stress markers. Notably, the combined SA+As treatments decreased MDA content by 35%–40% and the H₂O₂ content by 35%–42% relative to those under the arsenic alone, demonstrating that SA effectively alleviated arsenic-induced oxidative damage by limiting lipid peroxidation and reactive oxygen species accumulation.

**Table 2.** Effect of salicylic acid (SA) on oxidative stress markers malondialdehyde (MDA) and hydrogen peroxide (H₂O₂) in three wheat varieties (Anaaj-17, Dilkash-20, and Subhani-21) under arsenic (As) stress. Values are expressed as nmol/g FW for MDA and μmol/g FW for H₂O₂. Different letters indicate significant differences among treatments (*p* < 0.05).

MDA				H_2_O_2_		
Treatments	Anaaj-17 (nmol/g FW)	Dilkash-20 (nmol/g FW)	Subhani-21 (nmol/g FW)	Anaaj-17 (μmol/g FW)	Dilkash-20 (μmol/g FW)	Subhani-21 (μmol/g FW)
T0 (control)	6.25d	5.10d	7.05d	4.10d	3.25d	3.80d
T1 (SA1)	14.50c	12.80c	15.15c	13.00c	11.50c	12.50c
T2 (SA2)	16.10c	13.90c	17.00c	14.50c	12.80c	13.80c
T3 (As1)	55.40a	51.75a	53.90a	52.70a	50.15a	51.50a
T4 (As2)	62.80a	58.10a	60.50a	60.15a	57.50a	58.80a
T5 (SA1+As1)	33.50b	30.15b	31.85b	31.90b	29.50b	30.80b
T6 (SA1+As2)	37.90b	34.25b	35.95b	35.20b	32.80b	34.10b
T7 (SA2+As1)	35.00b	31.60b	33.25b	33.50b	31.10b	32.40b
T8 (SA2+As2)	39.50b	35.50b	37.50b	36.80b	34.40b	35.70b

Note: Means followed by different lowercase letters within a column are significantly different at *p* ≤ 0.05 according to LSD test.

## Discussion

Data evaluation revealed distinct physiological and anatomical responses of the wheat cultivars to salicylic acid (SA) and arsenic (As) treatments. SA exhibited growth-promoting effects, while As induced phytotoxicity. Plant height and leaf number increased under the SA treatment, contrasting with the decline observed under As stress. These findings align with previous reports showing the regulatory role of SA in promoting growth and development through enhanced cell elongation and division,^27^ whereas arsenic toxicity disrupts root and shoot development, leading to growth inhibition.^28^

SA treatment significantly improved fresh and dry biomass across all wheat varieties, while As caused substantial reductions. These results corroborate earlier findings that SA enhances biomass and yield by improving photosynthesis and metabolism,^29^ whereas As interferes with essential physiological processes, suppressing plant growth and productivity.^30^ The positive influence of SA could be attributed to its role in modulating the hormonal balance, enhancing antioxidant enzyme activity, and improving stress signaling pathways that collectively support biomass accumulation even under stress conditions.

Chlorophyll and relative water content (RWC) are key indicators of plant vitality and stress tolerance. In this study, significant variation in chlorophyll content was observed among wheat cultivars under different treatments. SA application, particularly at 1 mM, increased chlorophyll levels, indicating its role in stabilizing chloroplast membranes and promoting pigment biosynthesis. This finding is consistent with reports that SA enhances chlorophyll biosynthesis and photosynthetic efficiency under stress.^31^ Conversely, As exposure drastically reduces the chlorophyll content, confirming that heavy metals interfere with chlorophyll biosynthesis and degrade photosynthetic pigments.^32^ This reduction is likely due to reactive oxygen species (ROS) generation and impairment of chlorophyll synthesis pathways.^33^^,^^34^

Interestingly, combined treatment with 1 mM SA and 300 μM As mitigated the inhibitory effects of As on the chlorophyll content. This synergistic effect may result from SA's antioxidant capacity, which reduces oxidative damage and preserves pigment integrity.^35^ The improved photosynthetic pigment stability under the combined treatment highlights SA's potential as a protective signaling molecule that enhances plant resilience to metal toxicity.

RWC analysis further confirmed the positive impact of SA on the plant water balance. Increased RWC under SA application suggests improved water retention and turgor maintenance, particularly at higher concentrations. These findings are in accordance with those of Miura and Tada,^16^ who reported that SA modulates stomatal function and osmotic adjustment, leading to enhanced drought and stress tolerance. Conversely, As exposure reduced RWC, likely due to its negative impact on aquaporin function and guard cell signaling, resulting in reduced water uptake and stomatal closure.^36^ Co-application of SA with As alleviated this decline, indicating that SA mitigates water deficit effects by maintaining membrane stability and regulating osmolyte accumulation.

These results indicate that SA enhances the plant's antioxidant potential by activating enzymatic defense systems such as SOD and POD, thereby scavenging reactive oxygen species (ROS) and protecting its cellular structures. These findings are consistent with previous studies showing that SA alleviates heavy metal-induced oxidative stress in wheat and other crops by acting as a signaling molecule to modulate stress-responsive pathways. The enhanced antioxidant capacity observed in our study under combined SA and arsenic treatments further confirms the critical role of SA in maintaining redox homeostasis and improving plant tolerance under abiotic stress conditions.^37^^,^^38^

These anatomical observations further substantiate these physiological responses. SA treatment enhanced leaf epidermal thickness, vascular bundle development, and mesophyll density, suggesting improved structural defense and photosynthetic efficiency. Increased upper and lower epidermis (UE and LE) thickness under SA treatments supports previous findings that SA strengthens epidermal tissues to prevent excessive transpiration and pathogen invasion.^21^^,^^39^ Conversely, As exposure reduced epidermal thickness, reflecting cellular damage, and impaired structural integrity.^40^^-^^42^

Vascular bundle thickness was also enhanced by SA but reduced under As stress. The stimulatory effect of SA may be linked to its role in xylem and phloem differentiation and secondary cell wall formation, which improve water and nutrient transport.^43^ In contrast, As interferes with vascular differentiation, causing deformation and restricted conductivity. Similarly, SA treatment increased xylem and phloem thickness, which is consistent with the findings of Rivas-San Vicente and Plasencia,^44^ who demonstrated the involvement of SA in vascular tissue differentiation and defense signaling.

Mesophyll thickness increased under SA treatment, reflecting improved photosynthetic tissue development and chloroplast function.^45^ On the other hand, As stress led to a reduction in mesophyll layers, which can be attributed to chloroplast disorganization and impaired photosynthetic activity.^46^ These structural modifications under SA application provide a strong anatomical basis for enhanced physiological performance under stress conditions.

In roots, the anatomical parameters showed a consistent pattern with the leaf observations. SA treatments enhanced root exodermis, endodermis, cortex, and vascular tissue thickness, while As exposure had detrimental effects. SA-induced exodermis thickening strengthens mechanical support and improves selective water and ion uptake, which aligns with findings that SA enhances root protection against environmental stresses.^47^ In contrast, As treatments reduced exodermal thickness, indicating tissue degradation and a weakened root structure.^9^

The endodermis, a critical barrier regulating ion transport, showed increased thickness under SA, implying improved selectivity and enhanced stress tolerance.^48^^,^^49^ As exposure, however, led to thinning of this layer, likely due to cell wall disintegration and reduced nutrient transport.^50^ SA also promoted cortical thickening, indicating increased storage capacity and structural rigidity, which is consistent with reports of SA-induced lignification and cortical cell expansion.^51^ Conversely, As reduced cortex thickness, reflecting disrupted cell division and metabolic inhibition.^52^

The observed improvement in xylem and phloem organization under SA treatment suggests enhanced water and nutrient conductivity, which is crucial for stress adaptation. SA likely promotes vascular differentiation and lignin biosynthesis, ensuring effective translocation and defense against oxidative stress. Conversely, As disrupts vascular development, limiting nutrient flow and overall root vitality.^53^

Overall, these findings confirm that SA mitigates As-induced toxicity by maintaining leaf and root anatomical integrity, stabilizing chlorophyll and water relations, and enhancing vascular development. These physiological and structural improvements explain the observed differences in tolerance among wheat cultivars. Variation in cultivar response may arise from inherent genetic diversity influencing SA perception, transport, and signaling pathways that regulate antioxidant and defense responses.

From an agronomic perspective, these findings underscore the practicality of SA application as a low-cost and effective strategy for mitigating heavy metal stress. Foliar or soil application of SA at concentrations between 0.5–1 mM is feasible under field conditions due to its affordability, availability, and compatibility with standard spraying techniques. Integrating SA into stress management programs could improve crop resilience, sustain yield, and potentially maintain grain quality under contaminated conditions. However, while this study highlights cultivar-specific variations and structural mechanisms underlying stress mitigation, it does not explore the molecular or genetic factors responsible for these responses. Future investigations incorporating transcriptomic or proteomic profiling and long-term field trials focusing on SA dosage, application timing, and its effect on yield and grain quality will be valuable. Such studies will provide mechanistic insight into SA-mediated signaling and enable the development of SA-based strategies for sustainable wheat production under heavy metal stress.

## Conclusion

The positive effects of salicylic acid on seedlings were established by showing increased effects on plant growth, photosynthesis efficiency, and anatomical structures of the seedlings under salicylic acid treatment compared to untreated control plants and plants treated with arsenic. The physiological traits of wheat plants grown from seeds treated with salicylic acid enhanced the growth characteristics of the seedlings, as they elicited salicylic acid to reduce the impacts of arsenic on their growth. Aging was confirmed by physiological analysis, which presented increased chlorophyll and carotenoid concentrations, well-watered capacity, and accumulating total proteins in salicylic acid-treated seedlings and elucidated improved stress tolerance. Morphological analysis also aided in identifying the changes in the root and leaf structure in the seedlings treated with salicylic acid, supporting the positive effects of salicylic acid on root growth and structural reinforcement. These observations highlight the importance of using salicylic acid as a potential tool for effectively protecting agricultural plants in areas contaminated with arsenic and develop a basis for further investigations to determine the molecular background of the actions mentioned above of salicylic acid in wheat plants.

## Supplementary Material

Supplementary material Supplementry_data_usva_clean.docx

## Data Availability

The data and materials will be made available from the corresponding author upon request.
