# Supplementary material for: Salicylic acid mitigates arsenic-induced toxicity in wheat by enhancing growth and anatomical traits
Source: Plant Signal Behav. 2026 Feb 10;21(1):2626633. doi: 10.1080/15592324.2026.2626633 (PMC12928664; doi:10.1080/15592324.2026.2626633)
Supplement: Supplementary material — Supplementry_data_usva_clean.docx [file KPSB_A_2626633_SM9637.docx]

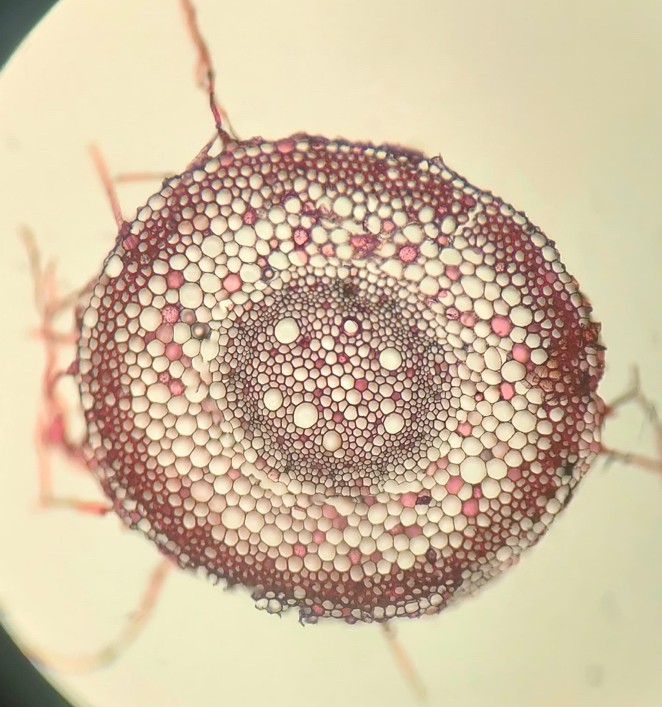

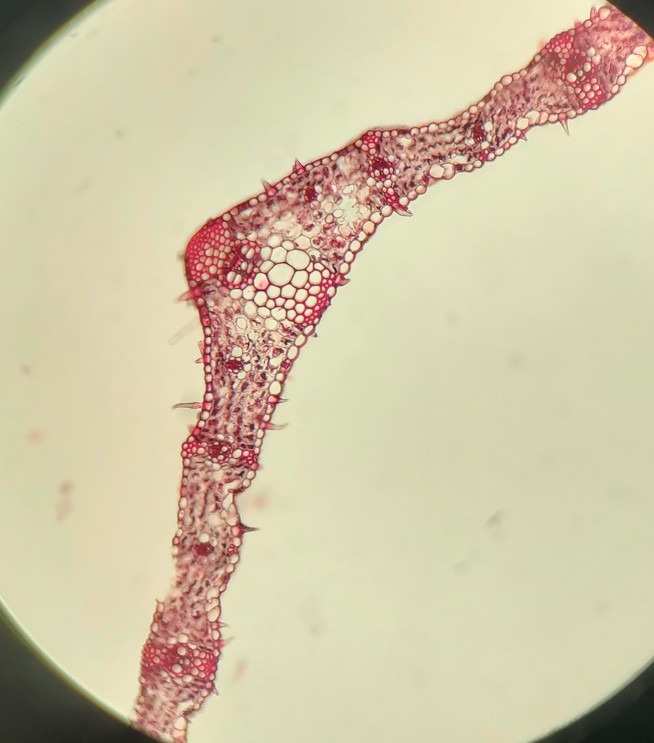


Plate 3.5 labelled anatomical sections of root and leaf of wheat plant.

**Leaf**

**Root**

Phloem

Lamina

Cortex

Metaxylem

Epidermis

Sclerenchyma layer

Phloem

Early metaxylem

Endodermis

Root cortical aerenchyma

Exodermis

Epidermis

**Supplementary Figure S1**
